# Structural complexity of simple Fe_2_O_3_ at high pressures and temperatures

**DOI:** 10.1038/ncomms10661

**Published:** 2016-02-11

**Authors:** E. Bykova, L. Dubrovinsky, N. Dubrovinskaia, M. Bykov, C. McCammon, S. V. Ovsyannikov, H. -P. Liermann, I. Kupenko, A. I. Chumakov, R. Rüffer, M. Hanfland, V. Prakapenka

**Affiliations:** 1 Bayerisches Geoinstitut, University of Bayreuth, Universitaetsstrasse 30, D-95447 Bayreuth, Germany; 2 Laboratory of Crystallography, University of Bayreuth, Universitaetsstrasse 30, D-95447 Bayreuth, Germany; 3 Photon Sciences, Deutsches Elektronen-Synchrotron, Notkestrasse 85, D-22607 Hamburg, Germany; 4 European Synchrotron Radiation Facility, 71 avenue des Martyrs, Grenoble F-38000, France; 5 Center for Advanced Radiation Sources, University of Chicago, 9700 South Cass Avenue, Illinois, Argonne 60437, USA

## Abstract

Although chemically very simple, Fe_2_O_3_ is known to undergo a series of enigmatic structural, electronic and magnetic transformations at high pressures and high temperatures. So far, these transformations have neither been correctly described nor understood because of the lack of structural data. Here we report a systematic investigation of the behaviour of Fe_2_O_3_ at pressures over 100 GPa and temperatures above 2,500 K employing single crystal X-ray diffraction and synchrotron Mössbauer source spectroscopy. Crystal chemical analysis of structures presented here and known Fe(II, III) oxides shows their fundamental relationships and that they can be described by the homologous series *n*FeO·*m*Fe_2_O_3_. Decomposition of Fe_2_O_3_ and Fe_3_O_4_ observed at pressures above 60 GPa and temperatures of 2,000 K leads to crystallization of unusual Fe_5_O_7_ and Fe_25_O_32_ phases with release of oxygen. Our findings suggest that mixed-valence iron oxides may play a significant role in oxygen cycling between earth reservoirs.

## Abstract (web summary)

Fe_2_O_3_ is known to undergo a series of structural, electronic and magnetic transformations at high pressures and temperatures but these are poorly understood due to a lack of structural data. Here, the authors perform experiments to elucidate the transformations and relationships between them.

## Graphical abstract

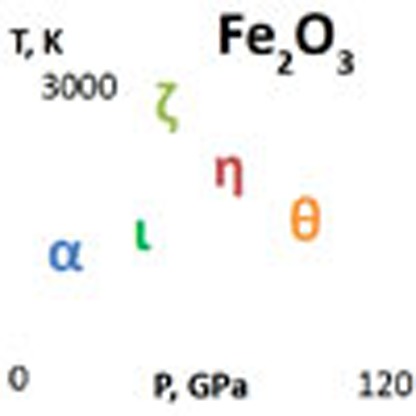

The structures, properties and high-pressure behavior of corundum-type oxides have been extensively investigated because of their wide variety of elastic, electrical and magnetic properties and importance in earth sciences and technology^1^,^2^,^3^. High-pressure studies of hematite, α-Fe_2_O_3_ (Fig. 1a), have attracted special attention due to their geophysical interest and the unclear role of Fe^3+^ in the nature and dynamics of the earth's lower mantle^1^,^2^. Particular attention has been focused on elucidating the nature of phase transition(s) and the structure of the high-pressure phase of hematite observed above ∼50 GPa (refs. 1,4,5,6,7,8,9,10,11,12). For this phase two structures have been proposed by different groups: Rh_2_O_3_-II-type (space group *Pbcn*, no. #60) and GdFeO_3_-perovskite-type (space group *Pbnm*, no. #62) structures^4^,^7^. While Mössbauer spectroscopic and resistivity measurements clearly demonstrate the importance of electronic changes in Fe^3+^ and seem to support the Rh_2_O_3_-II-type structure^5^, powder diffraction data collected by various groups over several decades did not allow an unambiguous assignment of the structural type (see refs 4, 5, 7, 8 and references therein). Only recent single-crystal high-*P*,*T* diffraction data^12^ were able to solve this challenge; they demonstrated that the Rh_2_O_3_-II-type phase of Fe_2_O_3_ (which we refer to below as 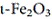, Fig. 1b) forms upon laser heating at pressures above ∼40 GPa; whereas, compression of hematite at ambient temperature to over ∼50 GPa results in the formation of a phase with distorted GdFeO_3_-perovskite-type, dPv ζ-Fe_2_O_3_, structure (Fig. 1c). Experiments in laser-heated diamond anvil cells (DACs) revealed the formation of a CaIrO_3_-type phase (‘post-perovskite' (PPv) η-Fe_2_O_3_, Fig. 1d) at pressures above ∼60 GPa (refs. 1,9,12,13). However, the behaviour of this phase under compression is not well studied. The phase diagram of Fe_2_O_3_ in the megabar pressure range is incomplete and the data are often conflicting^1^,^9^,^10^,^13^.

In order to study the high-pressure high-temperature (HPHT) behaviour of ferric iron (Fe^3+^) oxide we apply the complementary methods of single crystal X-ray diffraction in laser-heated DACs and synchrotron Mössbauer source (SMS) spectroscopy (see Methods section). We observe hitherto unknown Fe–O phases, show the results of their structure solution and refinement, and characterize the pressure–temperature conditions, at which different Fe_2_O_3_ polymorphs occur. Crystal chemical analysis of the new structures and known Fe(II, III) oxides reveals their fundamental relationships as members of the homologous series *n*FeO·*m*Fe_2_O_3_. We observe that at pressures above 60 GPa and at high temperatures (that is, at conditions of the earth's lower mantle), Fe_2_O_3_ decomposes with release of oxygen. The same phenomenon is observed for Fe_3_O_4_. Our results indicate that mixed-valence iron oxides may play a significant role in oxygen cycling between the earth's atmosphere and mantle.

## Results

### Structural transformations in Fe_2_O_3_

**Figure 1:**
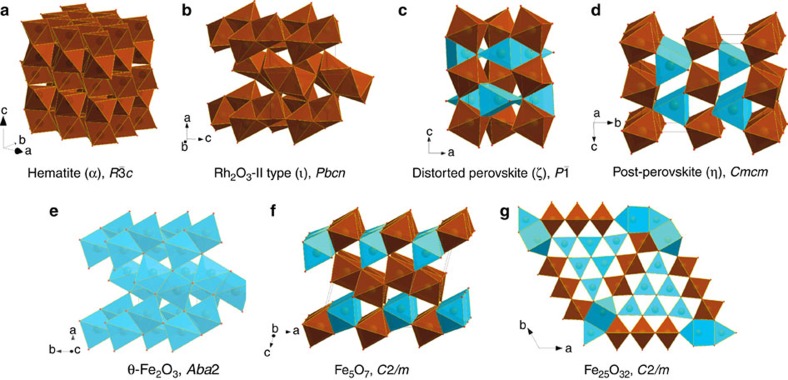
Crystal structures of iron oxide phases studied in the present work. Building blocks are octahedra (brown) and trigonal prisms (blue). The prisms in Fe_5_O_7_, Fe_25_O_32_ and η-Fe_2_O_3_ have one or two additional apices. Hematite (**a**) consists of FeO6 octahedra connected in a corundum-like motif, namely each octahedron connects with three neighbours via edges in honeycomb layers, and layers are interconnected through common triangular faces of octahedra. The 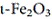 structure (**b**) is built of only FeO6 octahedra but each two octahedra are connected through a common triangular face; such units pack in a herringbone pattern and layers pack with a shift along the *c*-direction having common edges. In distorted perovskite ζ-Fe_2_O_3_ (**c**) octahedra connect through common vertices and prisms share only common edges. θ-Fe_2_O_3_ (**e**) adopts the packing motif from 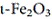 but instead of octahedra it consists of FeO6 prisms. Post-perovskite (**d**) and Fe_5_O_7_ (**f**) are members of the homologous series *n*FeO·*m*Fe_2_O_3_ (see also Fig. 4), where prisms are connected through common triangular faces, while octahedra connect only via shared edges. In addition to triangular face-shared prisms and edge-shared octahedra, Fe_25_O_32_ (**g**) has edge-shared one-capped prisms; therefore it belongs neither to the homologous series nor adopts any other known structural motif.

**Figure 2:**
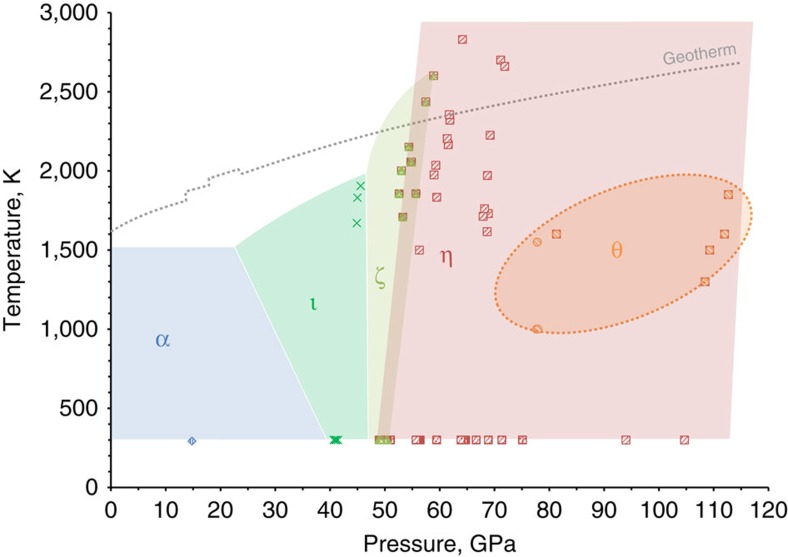
Transformational phase diagram of Fe_2_O_3_. (**a**) ⋄-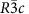 hematite (α-Fe_2_O_3_), Δ-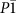 distorted perovskite (ζ-Fe_2_O_3_), ○-*Aba*2 (θ-Fe_2_O_3_, probably metastable), □-*Cmcm* post-perovskite (η-Fe_2_O_3_) and × -Rh_2_O_3_-II type phase (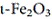). The boundary between hematite α-Fe_2_O_3_ and 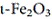 is defined according to ref. 10. The geotherm is defined according to refs 39, 40.

In agreement with previous studies^4^,^5^,^7^,^8^,^12^, our cold compression experiments on hematite single crystals to 54(1) GPa result in a transition to the ζ-Fe_2_O_3_ phase manifested by a ∼8.4% volume discontinuity (Supplementary Fig. 1). Although in earlier work we indexed the diffraction pattern of ζ-Fe_2_O_3_ in a monoclinic unit cell^12^, the new extended dataset acquired in the present work showed that the structure is in fact triclinic (see Supplementary Note 1 for details), similar to Mn_2_O_3_ (ref. 14). An insufficient number of independent reflections prevented structural refinement of ζ-Fe_2_O_3_ in triclinic symmetry, so we used a monoclinic model^12^ to qualitatively constrain the atomic arrangement in ζ-Fe_2_O_3_. Upon further pressure increase from 54(1) to 67(1) GPa, we observed a reduction in the splitting of reflections, indicating an increase in symmetry. The structure of ζ-Fe_2_O_3_ thus becomes closer to that of GdFeO_3_-type-perovskite (Supplementary Fig. 2b). At 67(1) GPa a small drop in the unit cell volume (∼1.7%) manifests the next transformation to the θ-Fe_2_O_3_ phase (Fig. 1e) with orthorhombic symmetry (space group *Aba*2, no. #41, *a*=4.608(7), *b*=4.730(4), *c*=6.682(18) Å (Supplementary Table 1)). On compression at ambient temperature θ-Fe_2_O_3_ can be observed to at least 100 GPa (Supplementary Fig. 1). The transformational *P*–*T* diagram for Fe_2_O_3_ is given in Fig. 2.

During *in situ* laser heating of θ-Fe_2_O_3_ between ∼1,000 and 1,550(50) K at 78(2) GPa, we observed no evidence of a phase transformation. The absence of transformations may either be evidence that θ-Fe_2_O_3_ is stable at these conditions or an indication that higher temperatures are required to overcome kinetic barriers to further structural transitions. Indeed, heating at 1,600(50) K results in the formation of post-perovskite type η-Fe_2_O_3_ coexisting with θ-Fe_2_O_3_. Both phases (θ-Fe_2_O_3_ and η-Fe_2_O_3_) were observed *in situ* simultaneously upon heating to 1,850(50) K at pressures up to 113(1) GPa. However, temperature-quenched products contained only η-Fe_2_O_3_ (Fig. 2). Once synthesized, η-Fe_2_O_3_ may be preserved at ambient temperature down to at least 26 GPa. At lower pressures it transforms back to hematite (see Figs 1 and 2 for structures and phase relations). Moderate heating to ∼2,000 K at pressures of about 50 GPa provokes a transition to the dPv ζ-Fe_2_O_3_ phase. Decompression of ζ-Fe_2_O_3_ or η-Fe_2_O_3_ to 41(1) GPa with heating at 1,800(100) K results in growth of Rh_2_O_3_-II type 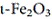 (Supplementary Fig. 1, Supplementary Table 1). Interestingly, 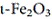 was synthesized earlier^10^,^11^ from hematite, thus bracketing the possible *P–T* stability field of the phase (Fig. 2).

### Electronic transformations in Fe_2_O_3_

**Figure 3:**
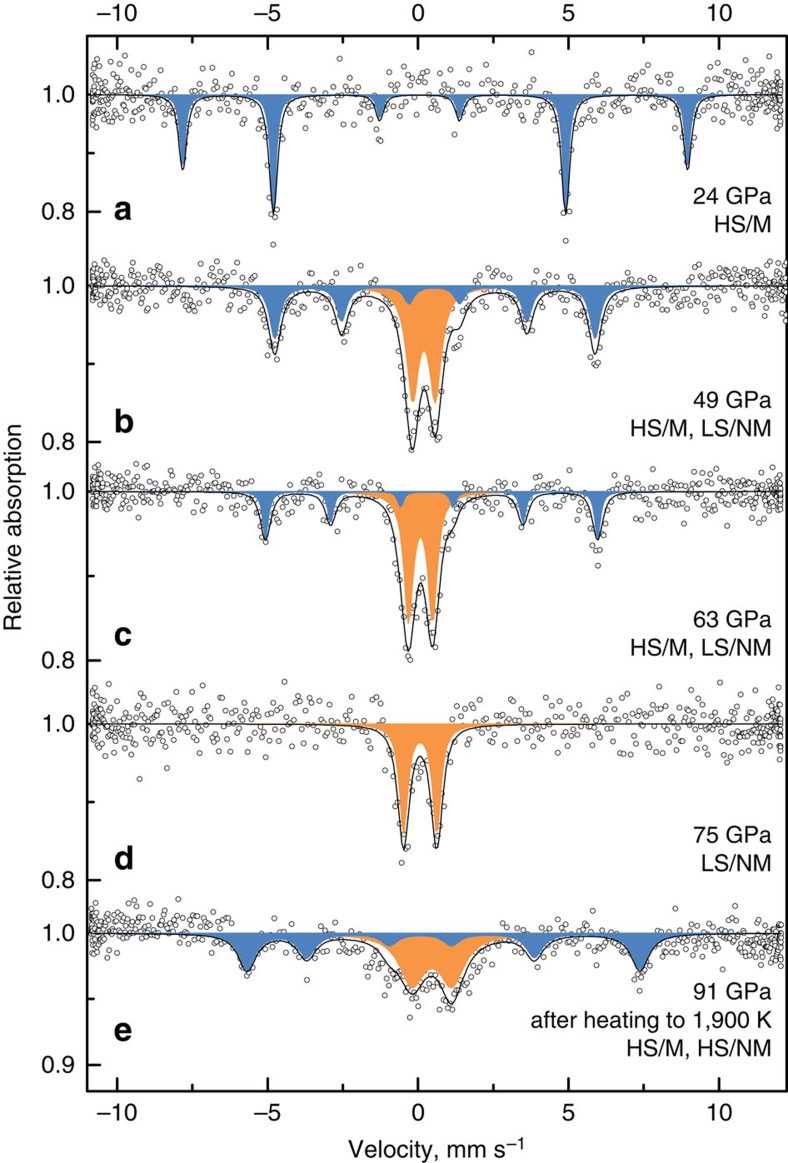
Evolution of SMS spectra of Fe_2_O_3_. Spectra collected during compression (**a**–**d**) and after heating (**e**). In hematite (**a**) iron atoms have a HS state (at ∼24 GPa CS=0.306(4) mm s^-1^), and spectra are split due to magnetic ordering (M). After the first transition at 49 GPa (**b**) a new non-magnetic (NM) component appears with CS of 0.074(5) mm s^-1^ corresponding to a LS state. During further compression a fraction of the magnetic component decreases (**c**) and it disappears completely after the second transition to the θ-Fe_2_O_3_ phase (**d**) that has only one non-magnetic position of LS iron atoms in the crystal structure. After heating above 1,600(50) K (**e**) a transformation to η-Fe_2_O_3_ occurs. The crystal structure has two HS-iron positions (both CS are ∼0.45 mm s^-1^), where one position is magnetically ordered and the other is non-magnetic.

The sequence of phase transitions in Fe_2_O_3_ in the megabar pressure range and temperatures up to about 2,500 K (Fig. 2) can be neatly rationalized through the variation of molar volumes of the phases observed as a function of pressure (Supplementary Fig. 1), complemented by the corresponding SMS spectroscopy data (Fig. 3). The bulk modulus of hematite, 219(7) GPa, is in good agreement with previous studies^15^ and at 67 GPa it reaches ∼392(10) GPa, whereas the bulk modulus of ζ-Fe_2_O_3_ at 54 GPa is substantially lower, 320(18) GPa. Such a large drop of bulk modulus (∼18%) associated with a large reduction of molar volume (∼8.4%) is very unusual and is likely caused by changes in the electronic state of Fe^3+^. The Mössbauer spectrum of ζ-Fe_2_O_3_ collected immediately after the transition at ∼50 GPa shows two components (Fig. 3b), a magnetic sextet having centre shift (CS) of 0.424(7) mm s^−1^ corresponding to the high-spin (HS) state of Fe^3+^, and a doublet (CS=0.074(5) mm s^−1^) with hyperfine parameters characteristic for low-spin (LS) Fe^3+^ in an octahedral oxygen environment^16^. The relative abundance of the components is ∼1:1, as expected for the perovskite-type structure of ζ-Fe_2_O_3_ with HS-Fe^3+^ located in large bipolar prisms and LS-Fe^3+^ in smaller octahedra (Fig. 1c). Upon further compression of ζ-Fe_2_O_3_ the amount of HS-Fe^3+^ decreases (Fig. 3c), which explains the anomalously high compressibility of this phase.

Transformation to θ-Fe_2_O_3_ is associated with a small decrease of molar volume (∼1.7%) and an increase of bulk modulus as expected (418(11) GPa for θ-Fe_2_O_3_ at 67 GPa compared with 371(20) GPa for ζ-Fe_2_O_3_ at 70 GPa) (Supplementary Fig. 1). The Mössbauer spectrum of θ-Fe_2_O_3_ (Fig. 3d) shows that all Fe^3+^ is in the LS state and there is only one type of iron atom in the crystal structure in accordance with the single crystal X-ray diffraction data (Fig. 1e).

Heating of θ-Fe_2_O_3_ above 1,600 K at pressures above 70 GPa resulted in partial or complete transformation into CaIrO_3_-PPv-type η-Fe_2_O_3_ (Fig. 2). The Mössbauer spectrum of pure η-Fe_2_O_3_ at 91(2) GPa (Fig. 3e) contains two components (a magnetically ordered sextet and a paramagnetic doublet) with equal abundances and almost equal CS (∼0.45 mm/s) corresponding to HS-Fe^3+^. Within the accuracy of our X-ray diffraction data the molar volumes of θ-Fe_2_O_3_ and as-synthesized η-Fe_2_O_3_ are indistinguishable (Supplementary Fig. 1), suggesting that the atomic packing density increase in the CaIrO_3_-PPv-type η-Fe_2_O_3_ structure compensates the difference in ionic radii of HS and LS Fe^3+^ ions in the ζ-Fe_2_O_3_ structure. Note that Shim *et al*.^1^ also reported magnetic ordering in η-Fe_2_O_3_ based on nuclear forward scattering measurements. One of the magnetic sites described by the authors^1^ has hyperfine parameters close to those that we observed; however, the second non-magnetic component in the nuclear forward scattering spectra was not identified in ref. 1.

### Thermal stability of Fe_2_O_3_

The behaviour of η-Fe_2_O_3_ under heating is rather remarkable. First, we noted that its unit cell volume increases by up to 1% upon laser heating to about 2,000 K at ∼56 and 64 GPa. (Supplementary Fig. 3). Second, after heating for a few seconds to 2,700–3,000 K and 71 GPa we observed the immediate appearance of new sharp spots in the diffraction pattern. The peaks were indexed in the *C*2/*m* space group and the structure solution using direct methods identified the phase as a novel mixed-valence iron oxide with stoichiometry Fe_5_O_7_ (FeO·2Fe_2_O_3_) (Fig. 1f, Supplementary Table 2). Visual observations (particularly preservation of the shape of the samples upon heating) and careful analysis of diffraction patterns (absence of diffuse scattering) verify that samples were not melted in experiments where Fe_5_O_7_ was synthesized. The phase is preserved on decompression down to at least 41(1) GPa. Thus, we explain our observations as a continuous loss of oxygen by η-Fe_2_O_3_ upon heating at moderate temperatures and pressures above ∼60 GPa according to the reaction η-Fe_2_O_3_→η-Fe_2_O_3−δ_+0.5δ·O_2_. Note that a similar process is well known for perovskites^17^ and other oxides^18^. The reaction is accompanied by a partial reduction of Fe^3+^ to larger-sized Fe^2+^ that consequently increases the unit cell volume. Upon heating at sufficiently high temperature (above ∼2,700 K), the oxygen deficiency in η-Fe_2_O_3_ reaches a critical limit and provokes a reconstructive phase transition resulting in the formation of the mixed-valence iron oxide Fe_5_O_7_: 5η-Fe_2_O_3_→2Fe_5_O_7_+0.5O_2_. From both X-ray diffraction and Raman spectroscopy we did not find any evidence to suggest involvement of carbon from the diamond anvils in the chemical reactions. Indeed this was not expected, because at the HPHT conditions of our experiments carbon and oxygen do not react^19^. Mössbauer experiments show that laser heating of Fe_2_O_3_ at pressures above 80 GPa leads to formation of phases containing iron with hyperfine parameters characteristic of a mixed valence state (Supplementary Fig. 4).

**Figure 4:**
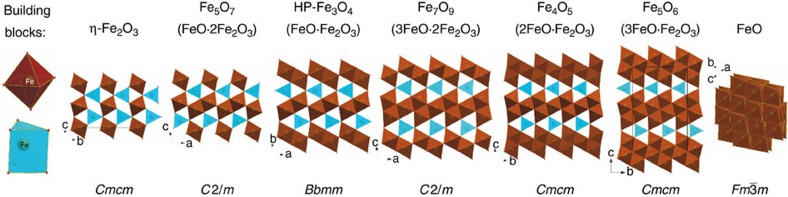
Homologous series of iron oxides described by the common formula *n*FeO·*m*Fe_2_O_3_. The structures may be described as assembled from two building blocks, FeO6 octahedra and trigonal prisms (prisms could be two-capped but they are not shown for simplicity). Prisms connect to each other through triangular faces, while octahedra share edges, so that they form parallel columns of face-shared prisms and edge-shared octahedra arranged in different motifs as seen in the figure with structures viewed from the top of the columns. Increasing Fe^2+^ content favours octahedral packing over mixed octahedral and prismatic packing. This requires denser packing of FeO6 octahedra and as a result columns of octahedra condense in slabs by sharing common edges. In particular, η-Fe_2_O_3_ has ordinary columns of prisms and octahedra with a chequerboard-like arrangement; Fe_5_O_7_ has ordinary and doubled columns of octahedra; the HP-Fe_3_O_4_ (ref. 41) possesses only doubled columns; Fe_7_O_9_ (ICSD reference number CSD-430601)^42^ has doubled columns and tripled columns organized in zigzag slabs; Fe_4_O_5_ (ref. 20) possesses only tripled and Fe_5_O_6_ (ref. 21) only quadruple zigzag slabs. The end-member of the homologous series wüstite (FeO) consists of octahedra with a maximum (12) number of edge-shared neighbours.

Similarities in the crystal structures of η-Fe_2_O_3_, Fe_5_O_7_, high-pressure polymorph of Fe_3_O_4_ (HP-Fe_3_O_4_, space group *Bbmm*, no. #63, Supplementary Tables 1 and 2), and the recently discovered Fe_4_O_5_ (ref. 20) and Fe_5_O_6_ (ref. 21) (Fig. 4) demonstrate^22^ that iron oxide phases form a homologous series *n*FeO·*m*Fe_2_O_3_ (with wüstite, FeO and η-Fe_2_O_3_ as the end-members) and indicate that a mixed-valence state of iron may become crystal chemically important at high pressures and temperatures.

## Discussion

Our results demonstrate clearly the complex behaviour of iron oxide subjected to high pressures and temperatures and may have significant consequences for modelling of the earth's interior. Hematite is one of the major components of banded iron formations (BIFs) and ironstones, and these huge sedimentary rock formations occurring on all continents may reach up to several hundred meters in thickness and hundreds of kilometres in length. Deposited in the world's oceans, BIFs as part of the ocean floor are recycled into the earth's interior by subduction^2^,^23^ to depths extending possibly to the core–mantle boundary region^2^. Available experimental data^2^,^13^,^24^ suggest that iron oxides melt above the geotherm in the entire mantle and thus remain solid in slabs that are colder than the surrounding mantle. Even assuming a slow subduction rate of 1 cm per year with slabs reaching a depth of about 2,000 km in ∼200 Ma, this geological time is sufficient to influence only a few tens of meters of rocks beneath the BIF's surface. Thus the fate of iron oxides, a major component of subducted BIFs, depends on the pressures and temperatures (*P–T*), to which they are exposed. Upon subduction of BIFs into the lower mantle, hematite undergoes numerous phase transformations. At pressures above ∼60 GPa the HP phase η-Fe_2_O_3_ starts to decompose, producing oxygen. Moreover, experiments on Fe_3_O_4_, the second major component of BIFs, show that it also decomposes upon heating at pressures above ∼70 GPa, forming in particular the phase Fe_25_O_32_ (Fig. 1g, see also Supplementary Note 2). Based on estimates of the amount of BIFs subducted into the earth's mantle^2^ and that BIFs may consist of ∼50% Fe_2_O_3_ by volume, the amount of oxygen produced by the formation of Fe_5_O_7_ alone can be as high as 8–10 times the mass of oxygen in the modern atmosphere. Extrapolation of available data^25^ indicates that oxygen would be in the liquid state at geotherm temperatures. Since the oxygen fugacity of the lower mantle is expected to be low through equilibrium with metallic iron, an oxygen-rich fluid could locally oxidize surrounding material (particularly Fe^2+^ in ferropericlase as well as bridgmanite, and metallic iron in a (Fe, Ni)-metal phase^26^). Seismic tomography reveals pronounced complex heterogeneities in the lower mantle at depths of 1,500–2,000 km associated with subducted slabs^27^,^28^,^29^ and the presence of oxidized material may be a reason for these observations^30^. On the other hand, a low oxygen chemical activity at high pressure^19^,^31^,^32^ could prevent the immediate reaction of oxygen in the lower mantle or even in the transition zone, and instead allow an oxygen-rich fluid to pass to the upper mantle, thus shifting Fe^2+^/Fe^3+^ equilibria in silicate minerals and greatly raising the oxygen fugacity in this region. In any case, our study suggests the presence of an oxygen-rich fluid in the deep earth's interior that can significantly affect geochemical processes by changing oxidation states and mobilizing trace elements.

## Methods

### Sample preparation

Single crystals of α-Fe_2_O_3_ enriched with ^57^Fe (^57^Fe_2_O_3_) were grown by means of HPHT technique at 7 GPa and 800 °C in a 1,200-t Sumitomo press at Bayerisches Geoinstitut (Bayreuth, Germany). As a precursor, a 1:1 mixture of a powder of non-enriched hematite (α-Fe_2_O_3_) of 99.998% purity and a pure powder of ^57^Fe_2_O_3_ (96.64%-enriched) was used. Magnetite synthesis was performed in the same way at 9.5 GPa and 1,100 °C as described in ref. 33. Synthesis of non-enriched hematite single crystals was described in ref. 15.

Single crystals with an average size of 0.03 × 0.03 × 0.005 mm^3^ were pre-selected on a three-circle Bruker diffractometer equipped with a SMART APEX CCD detector and a high-brilliance Rigaku rotating anode (Rotor Flex FR-D, Mo-Kα radiation) with Osmic focusing X-ray optics.

Selected crystals together with small ruby chips (for pressure estimation) were loaded into BX90-type DACs^34^. Neon was used as a pressure transmitting medium loaded at Bayerisches Geoinstitut.

### X-ray diffraction

The single-crystal X-ray diffraction experiments were conducted on the ID09A beamline at the European synchrotron radiation facility (ESRF), Grenoble, France (MAR555 detector, *λ*=0.4126–0.4130 Å); on the 13-IDD beamline at the advanced photon source (APS), Chicago, USA (MAR165 CCD detector, *λ*=0.3344 Å); and on the extreme conditions beamline P02.2 at PETRA III, Hamburg, Germany (PerkinElmer XRD1621 flat panel detector, *λ*=0.2898–0.2902 Å). The X-ray spot size depended on the beamline settings and varied from 4 to 30 μm, where typically a smaller beam was used for laser heating experiments. A portable double-sided laser heating^35^ system was used for experiments on ID09A (ESRF) to collect *in situ* single-crystal X-ray diffraction. State-of-the art stationary double-side laser-heating set-up at IDD-13 (APS) has been used for temperature-quenched single-crystal X-ray diffraction. Crystals were completely ‘surrounded' by laser light and there were no measurable temperature gradients within the samples. In the case of prolonged heating experiments the temperature variation during the heating did not exceed ±100 K. Pressures were calculated from the positions of the X-ray diffraction lines of Ne (http://kantor.50webs.com/diffraction.htm). X-ray diffraction images were collected during continuous rotation of DACs typically from –40 to +40 on ω; while data collection experiments were performed by narrow 0.5–1° scanning of the same ω range. The crystallographic information is also available as Supplementary Data 1-7.

### Data analysis

Integration of the reflection intensities and absorption corrections were performed using CrysAlisPro software^36^. The structures were solved by the direct method and refined in the isotropic approximation by full matrix least-squares refinements using SHELXS and SHELXL software^37^, respectively.

### SMS spectroscopy

Energy-domain Mössbauer measurements were carried out at the nuclear resonance beamline ID18 at ESRF (see ref. 38 for more details).

## Additional information

**Accession codes:** The X-ray crystallographic coordinates for structures reported in this article have been deposited at the Inorganic Crystal Structure Database (ICSD) under deposition number CSD 430557–430563. These data can be obtained free of charge from FIZ Karlsruhe, 76344 Eggenstein-Leopoldshafen, Germany (fax: (+49)7247-808-666; e-mail: crysdata@fiz-karlsruhe.de) through the hyperlink ‘ https://www.fiz-karlsruhe.de/en/leistungen/kristallographie/kristallstrukturdepot/order-form-request-for-deposited-data.html'.

**How to cite this article:** Bykova, E. *et al*. Structural complexity of simple Fe_2_O_3_ at high pressures and temperatures. *Nat. Commun.* 7:10661 doi: 10.1038/ncomms10661 (2016).

## Supplementary Material

Supplementary Information Supplementary Figures 1-4, Supplementary Tables 1-2, Supplementary Notes 1-2 and Supplementary References.

Supplementary Data 1 Crystallographic information file of η-Fe_2_O_3_ at 64 GPa

Supplementary Data 2 Crystallographic information file of η-Fe_2_O_3_ at 74 GPa

Supplementary Data 3 Crystallographic information file of HP-Fe_3_O_4_ at 44 GPa

Supplementary Data 4 Crystallographic information file of Fe_5_O_7_ at 41 GPa

Supplementary Data 5 Crystallographic information file of Fe_25_O_32_ at 80 GPa

Supplementary Data 6 Crystallographic information file of θ-Fe_2_O_3_ at 74 GPa

Supplementary Data 7Crystallographic information file of 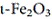 at 41 GPa
