# Supplementary material for: Structural complexity of simple Fe2O3 at high pressures and temperatures
Source: Nat Commun. 2016 Feb 11;7:10661. doi: 10.1038/ncomms10661 (PMC4753252; doi:10.1038/ncomms10661)
Supplement: Supplementary Information — Supplementary Figures 1-4, Supplementary Tables 1-2, Supplementary Notes 1-2 and Supplementary References. [file ncomms10661-s1.pdf]

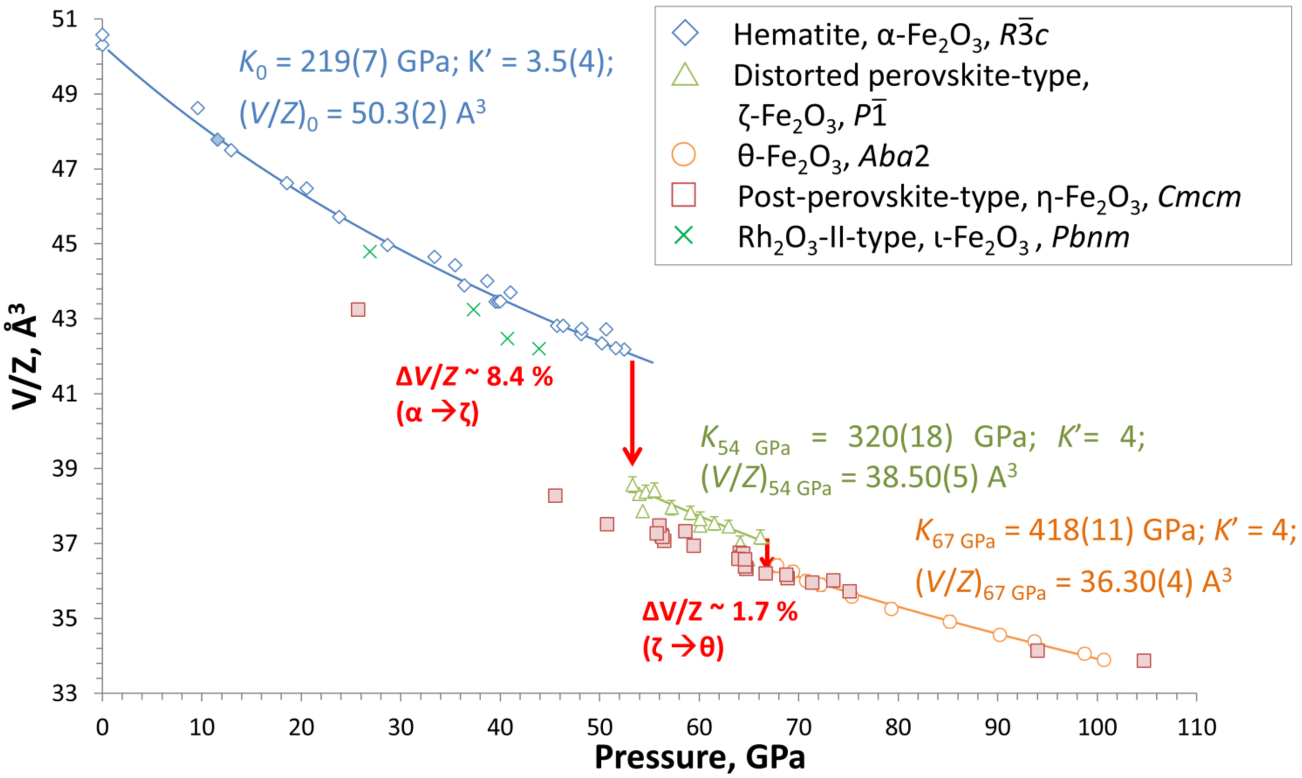


**Supplementary Figure 1**: ***P*-*V* plot for $Fe_2O_3$ summarized from current experimental results**. Unit cell volumes are normalized to the amount of structural units *Z*. Open symbols represent ambient-temperature experiments and solid symbols indicate samples subjected to laser heating. The volume relaxation of η-$Fe_2O_3$ under decompression shows apparent discontinuities after annealing at ~56 GPa and 64 GPa due to possible decomposition (see Supplementary Figure 3 for details). The volume of the high-temperature polymorph ι-$Fe_2O_3$ is lower than the volume of hematite at the identical pressure.

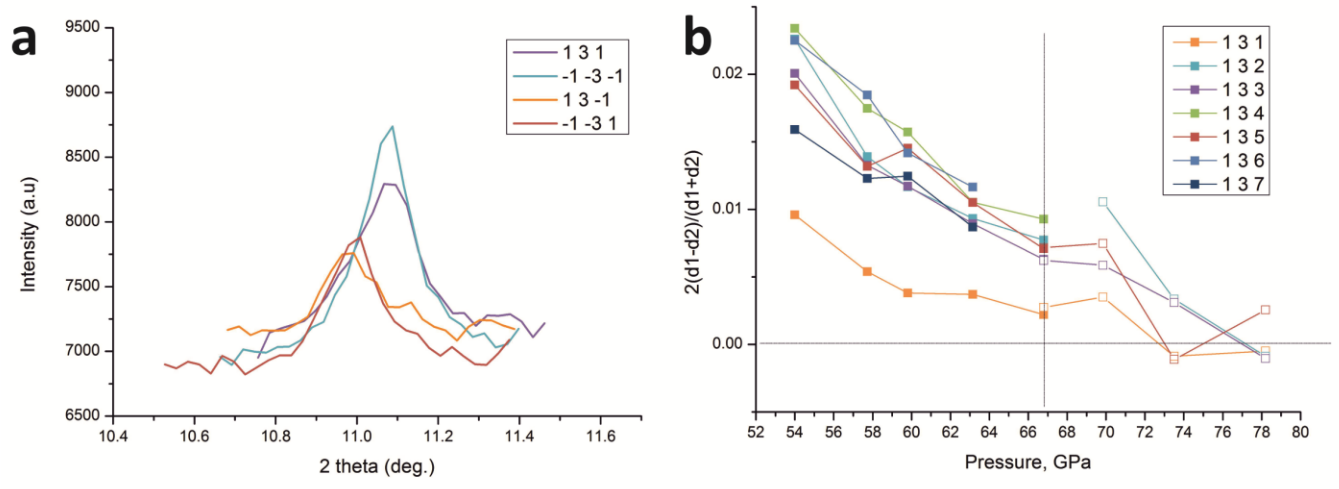


**Supplementary Figure 2: Verification of the crystal system for ζ-$Fe_2O_3$.** (*a*) We used one dimensional profiles of reflection 131 and its symmetrical equivalents in the hypothetical orthorhombic space group reconstructed from the wide image recorded at 60.0(7) GPa. While *d*-spacings for Friedel mates (131 and -1-3-1 / -1-31 and 13-1) show a perfect match, the first pair has smaller *d*-spacings (corresponding to $2\theta = 10.98°$) than the second one ($2\theta = 11.08°$). (*b*) The cumulative picture for reflections 13*l* ($l = 1 \ldots 7$) shows how the difference in *d*-spacings (normalized to corresponding sums) decreases under compression to half of its value before the phase transition to θ-$Fe_2O_3$ at 67 GPa (vertical dotted line) (see Supplementary Text for details).

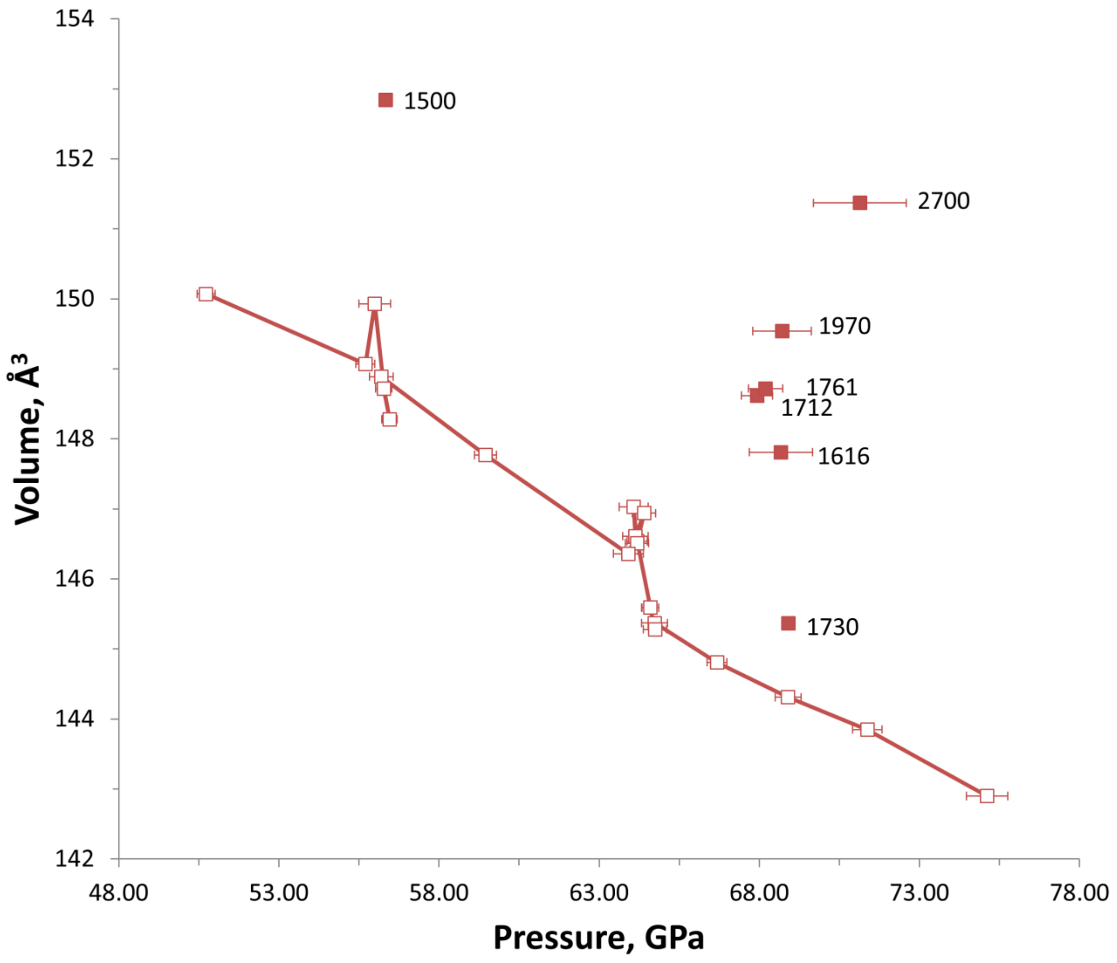


**Supplementary Figure 3: Unit cell volume of η-$Fe_2O_3$ under decompression and laser annealing**. The sequence of measurements goes from right to left. Numbers refer to the heating temperature with an average deviation of 100 K. Heating at ~56 GPa and 64 GPa provokes a unit cell volume increase of up to 1 % that is likely associated with the loss of oxygen by η-$Fe_2O_3$ and partial reduction of $Fe^{3+}$ to $Fe^{2+}$.

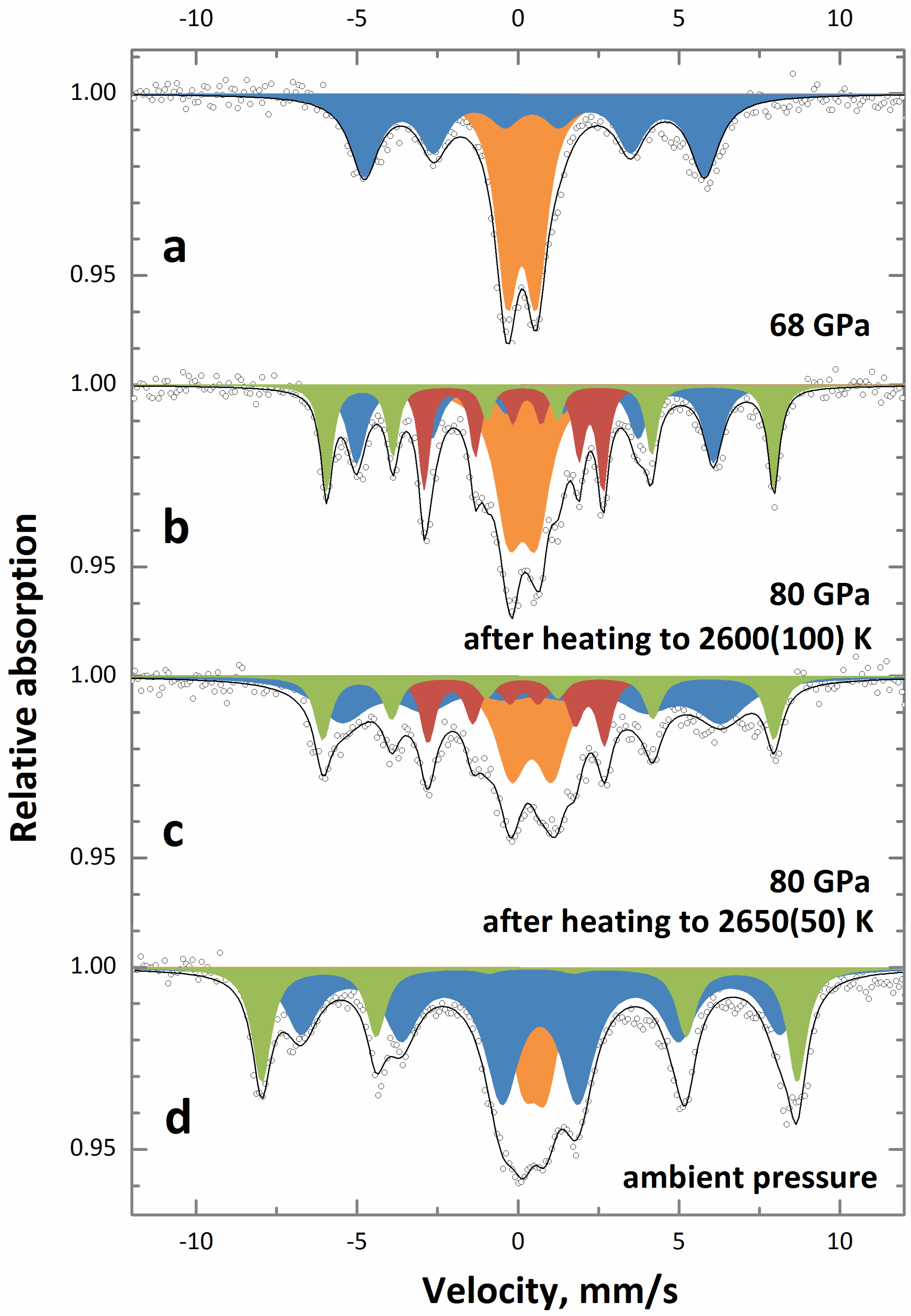

-10
-5
0
5
10
1.00
0.95
a
68 GPa
1.00
0.95
b
80 GPa
after heating to 2600(100) K
1.00
0.95
c
80 GPa
after heating to 2650(50) K
1.00
0.95
d
ambient pressure
Relative absorption
-10
-5
0
5
10
Velocity, mm/s

**Supplementary Figure 4. Examples of Mössbauer spectra collected using a $^{57}Co$ source from 50% enriched $^{57}Fe_2O_3$ compressed in Ar pressure transmitting medium and laser-heated in DAC**. (*a*) In ζ-$Fe_2O_3$ at 68(4) GPa iron atoms are in a high-spin magnetic state (with centre shift (CS) of 0.453(9) mm/s, blue sextet) and in low-spin non-magnetic state (CS = 0.113(4) mm/s, orange doublet). (*b*) After first heating during about 10 min at 2600(100) K pressure increased to 80(5) GPa and spectrum changed drastically: it consists of four components – three magnetic sextets (with CS (mm/s) 0.570(4), green; 0.516(8), blue; 0.074(4), red), and doublet (CS 0.154(7) mm/s, orange). (*c*) Several times subsequent heating at 2650(50) K with total duration over 40 min did not result in pressure changes (80(5) GPa), but Mössbauer spectra underwent some modifications und eventually became as shown in the figure: they consist of four components - three magnetic sextets (with CS (mm/s) 0.539(7), green; 0.498(8), blue; 0.065(7), red), and a doublet (CS 0.423(10) mm/s, orange). (*d*) After decompression the spectrum consists of three components, two magnetic sextets (with CS (mm/s) 0.382(6), green; 0.697(9), blue) and one doublet (CS 0.513(16) mm/s, orange). Note that the hyperfine parameters of the 'blue' component in (*d*) coincide almost exactly with those of $Fe^{2.67+}$ (mixed valence state "B-position") of magnetite at ambient conditions. The presence in the Mössbauer spectra (*b*, *c* ,*d*) of components with high CS (above 0.5 mm/s) demonstrates that laser heating leads to partial reduction of $Fe^{3+}$ and formation of $Fe^{2+}$ or, more probably, a mixed valence state of iron ions.

**Supplementary Table 1. Details of crystal structure refinements of high-pressure $Fe_2O_3$ phases**

| **Crystallographic data** | **θ-$Fe_2O_3$** | **η-$Fe_2O_3$** | **η-$Fe_2O_3$** | **ι-$Fe_2O_3$** |
|---|---|---|---|---|
| ***P*, *T* conditions of XRD experiment** | 73.8(7) GPa | 63.9(5) GPa, after annealing at 2200(100) K | 75.1(7) GPa after annealing at 1850(100) K | 40.7(3) GPa, after annealing at 1800(100) K |
| **Crystal system** | Orthorhombic | Orthorhombic | Orthorhombic | Orthorhombic |
| **Space group** | *Aba2* | *Cmcm* | *Cmcm* | *Pbcn* |
| ***a* (Å)** | 6.524(9) | 2.640(6) | 2.6393(7) | 7.062(10) |
| ***b* (Å)** | 4.702(3) | 8.639(9) | 8.5177(15) | 4.8108(13) |
| ***c* (Å)** | 4.603(7) | 6.414(14) | 6.358(2) | 5.0019(8) |
| **$V$ (Å$^3$)** | 141.2(3) | 146.3(5) | 142.93(7) | 169.9(2) |
| ***Z*** | 4 | 4 | 4 | 4 |
| ***F*(000)** | 304 | 304 | 304 | 304 |
| **Theta range for data collection (°)** | 4.03 to 15.13 | 2.68 to 11.10 | 3.76 to 10.96 | 3.08 to 10.82 |
| **Completeness to *d* = 0.8 Å, %** | 45.0 | 69.3 | 61.6 | 48.0 |
| **Index ranges** | $-7 < h < 4$, $-5 < k < 5$, $-4 < l < 5$ | $-2 < h < 2$, $-9 < k < 9$, $-6 < l < 5$ | $-2 < h < 2$, $-9 < k < 9$, $-5 < l < 5$ | $-2 < h < 4$, $-5 < k < 5$, $-5 < l < 5$ |
| **Reflections collected** | 54 | 93 | 81 | 206 |
| **Independent reflections / $R_{int}$** | 45 / 0.0329 | 53 / 0.0637 | 46 / 0.0372 | 61 / 0.1003 |
| **Refinement method** | Full matrix least squares on $F^2$ | | | |
| **Data / restraints / parameters** | 45 / 1 / 12 | 53 / 0 / 9 | 46 / 0 / 9 | 61 / 0 / 16 |
| **Goodness of fit on $F^2$** | 1.236 | 1.212 | 1.173 | 1.248 |
| **Final *R* indices [$I > 2\sigma(I)$]** | 0.0943 / 0.1669 | 0.0654 / 0.1635 | 0.0860 / 0.2101 | 0.0755 / 0.1942 |

| **Crystallographic data** | **θ-$Fe_2O_3$** | **η-$Fe_2O_3$** | **η-$Fe_2O_3$** | **ι-$Fe_2O_3$** |
|---|---|---|---|---|
| **$R_1$ / $wR_2$** | | | | |
| ***R* indices (all data) $R_1$ / $wR_2$** | 0.1058 / 0.1736 | 0.0910 / 0.1806 | 0.0909 / 0.2132 | 0.0847 / 0.1986 |
| **Largest diff. peak /hole (*e* / Å$^3$)** | 1.425 / -1.786 | 1.851 / -1.416 | 1.730 / -2.432 | 1.283 / -1.097 |
| **ICSD reference N** | 430557 | 430558 | 430559 | 430560 |

**Supplementary Table 2. Details of crystal structure refinements of $Fe_5O_7$ and $Fe_{25}O_{32}$**

| **Crystallographic data** | $Fe_5O_7$ | *HP*-$Fe_3O_4$ | $Fe_{25}O_{32}$ |
|---|---|---|---|
| ***P, T* conditions of XRD experiment** | 40.7(3) GPa, after annealing of $Fe_2O_3$ at 1800(100) K | 44.3(5) GPa, after annealing of $Fe_3O_4$ at 2350(100) K | 80.1(5) GPa, after annealing of $Fe_3O_4$ at 2950(100) K |
| **Crystal system** | Monoclinic | Orthorhombic | Hexagonal |
| **Space group** | *C*2/*m* | *Bbmm* | *P*-62*m* |
| ***a* (Å)** | 9.208(7) | 9.309(3) | 13.4275(16) |
| ***b* (Å)** | 2.7327(10) | 9.282(2) | 13.4275(16) |
| ***c* (Å)** | 8.270(5) | 2.6944(9) | 2.6289(4) |
| **α (°)** | 90 | 90 | 90 |
| **β (°)** | 105.50(8) | 90 | 90 |
| **γ (°)** | 90 | 90 | 120 |
| ***V* ($Å^3$)** | 200.5(2) | 232.80(11) | 410.48(12) |
| ***Z*** | 2 | 4 | 1 |
| ***F*(000)** | 372 | 440 | 906 |
| **Theta range for data collection (°)** | 2.77 to 10.78 | 3.57 to 11.05 | 3.32 to 15.47 |
| **Completeness to *d* = 0.8 Å, %** | 39.2 | 82.5 | 91.3 |
| **Index ranges** | -8 < *h* < 9, | -10 < *h* < 10, | -17 < *h* < 18, |
| | -2 < *k* < 2, | -11 < *k* < 10, | -18 < *k* < 18, |
| | -8 < *l* < 9 | -3 < *l* < 3 | -3 < *l* < 3 |
| **Reflections collected** | 106 | 517 | 2353 |
| **Independent reflections / $R_{int}$** | 72 / 0.0385 | 123 / 0.0544 | 487 / 0.1009 |
| **Refinement method** | Full matrix least squares on $F^2$ | | |
| **Data / restraints / parameters** | 72 / 0 / 18 | 123 / 0 / 17 | 487 / 0 / 50 |
| **Goodness of fit on $F^2$** | 1.056 | 1.253 | 1.086 |

| | | | |
|---|---|---|---|
| **Final *R* indices [*I* > 2σ(*I*)], $R_1$ / $wR_2$** | 0.0638 / 0.1541 | 0.0686 / 0.1649 | 0.0547 / 0.1257 |
| ***R* indices (all data), $R_1$ / $wR_2$** | 0.0673 / 0.1583 | 0.0691/ 0.1649 | 0.0805 / 0.1369 |
| **Largest diff. peak /hole (*e* / Å$^3$)** | 1.126 / -1.001 | 2.263 / -1.621 | 1.569 / -1.396 |
| **ICSD reference N** | 430563 | 430561 | 430562 |

**Supplementary Note 1. Verification of ζ-$Fe_2O_3$ crystal system**

Following our previous study[1] we verified the symmetry of the unit cell by inspecting *d*-spacings of those reflections that should have been equivalent in orthorhombic symmetry but differed in lower symmetries. It should be noted that previous powder diffraction studies were not able to resolve such differences due to the strong broadening of closely overlapping reflections. In single crystal XRD the overlapping problem is solved since the reflections are located at different places on the frames and their *d*-spacings could be measured separately.

The absence of orthorhombic symmetry can be clearly demonstrated by considering sets of candidate reflections 13*l* (*l* = 1, 2, … 7) with the following equivalents: 13*l*, -1-3*l*, 13-*l* and -1-3-*l*. Supplementary Figure 2a shows that the -1-31 and 13-1 reflections have larger *d*-spacings than the 131 and -1-3-1 ones. There is a perfect match in *d*-spacings between Friedel mates (131 and -1-3-1 / -1-31 and 13-1) indicating negligible effects of strain created by the DAC.

A verification of the monoclinic unit cell using a similar approach was hindered due to the lack of equivalent reflections for the particular symmetry defined by the specific orientation of the crystal in the DAC. Therefore we refined the unit cell based on the available reflections without symmetry constraints, i.e., a triclinic cell. As a result the alpha and beta angles show systematic scatter from 90° of about 0.4° while gamma varies by much less, ~ 0.1°. The overall scatter, representing the distortion from the orthorhombic perovskite type, is demonstrated in Supplementary Figure 2b, where it is expressed as the difference between *d*-spacings of the close reflections normalized to their sum.

**Supplementary Note 2. Notes on high pressure behavior of $Fe_3O_4$**

Single crystals of synthetic magnetite $Fe_3O_4$[2] were studied in double-side laser heated DACs at pressures up to 80 GPa and over 3000 K. Results of the study will be published elsewhere. Our single-crystal synchrotron XRD study of magnetite at ambient temperature confirmed the phase transition between 29 and 31 GPa and the $CaTi_2O_4$-type structure (space group *Bbmm*, No. #63) of the high-pressure *HP*-$Fe_3O_4$ phase (Fig. 4). Laser heating of *HP*-$Fe_3O_4$ at 2350(100) K and pressures up to 50 GPa does not result in any chemical or structural modifications (Supplementary Table 2). Evidence for the existence of the *HP*-$Fe_3O_4$ phase at even higher *P*,*T* conditions was found in our independent experiments on siderite ($FeCO_3$). By studying products of its decomposition after treatment at 70(1) GPa and 2400(100) K, we identified reflections in the XRD pattern that belong to *HP*-$Fe_3O_4$.

Upon heating at 80(1) GPa and 2950(100) we observed formation of a new phase with hexagonal unit cell (space group *P*-62*m*, No. #189). Structure solution from the single-crystal data revealed that the phase is a new iron oxide with composition $Fe_{25}O_{32}$ (Supplementary Table 2). Similar to iron oxides

belonging to the $n$FeO·$m$Fe$_2$O$_3$ homological series, $Fe_{25}O_{32}$ is a quasi-two-dimensional structure constructed of parallel columns of triangular face-shared prisms and edge-shared octahedra, but has in additional columns of edge-shared one-capped prisms (Fig. 1g).

## Supplementary References


1. Bykova, E. *et al.* Novel high pressure monoclinic $Fe_2O_3$ polymorph revealed by single-crystal synchrotron X-ray diffraction studies. *High Pressure Res.* **33,** 534–545 (2013).
2. Glazyrin, K. *et al.* Effect of high pressure on the crystal structure and electronic properties of magnetite below 25 GPa. *Am. Mineral.* **97,** 128–133 (2012).
